# The Process of Translating and Culturally Adapting a Digital Elder Abuse Intervention

**DOI:** 10.32996/ijtis.2022.2.1.2

**Published:** 2022-02-17

**Authors:** Maripaz Garcia, Dalia Pena-Solorzano, Chelsea Edwards, Fuad Abujarad

**Affiliations:** 1 Yale University, Department of Spanish and Portuguese, New Haven, CT, USA; 2 Yale New Haven Hospital, New Haven, CT, USA; 3 Research Support Coordinator, Yale School of Medicine, New Haven, CT, USA; 4 Yale School of Medicine, New Haven, CT, USA

**Keywords:** Elder abuse, translation, digital health, cultural adaptation, Hispanic/Latino, Spanish

## Abstract

Elder Abuse is a national public health problem affecting one in ten older adults. It is estimated that only 4% of cases are reported to authorities. Latino populations that reside in the U.S. are less likely to report abuse, and language barriers may limit access to resources and prevent seeking help. There is a need for tools and services to not only be translated but culturally adapted to ensure the integrity and comprehension of the translated product. We conducted an extensive literature review that informed our multi-step language translation and cross-cultural adaptation of the VOICES digital health elder abuse intervention from English to Spanish. This process involved a team of independent translators for an iterative, step-by-step approach that included synthesis and review at each step of the process. Translations were individually rated by the review team based on a 7-point Likert scale. The review team found the translations appropriate and highly satisfactory. Comparison of separate versions of translated items highlighted key linguistic variations and issues that informed the team when producing the final translated product. Challenges found during the translation process were categorized as a posteriori. Examples are included. Following a multi-step, iterative framework for the translation and cultural adaptation provided a highly accurate product. Involving multiple translators from varying backgrounds reduced the risk for translation bias and flagged cultural nuances that allowed the research team to identify areas that needed more attention and care. The product will be further culturally adapted with the help of the community via cognitive interviews with Spanish-speaking individuals relevant to the intervention’s intended target population before following up with a study to compare with the original findings of the intervention’s parent study.

## 1. Introduction

According to the World Health Organization (WHO), elder abuse is a “single or repeated act or lack of appropriate action, occurring within any relationship where there is an expectation of trust which causes harm or distress to an older person” (WHO, 2008, p. 15). Elder abuse is an ongoing health problem that affects approximately 10% of Americans aged 60 and older (Acierno et al., 2010; Swierenga et al., 2021). Giraldo-Rodríguez and Rosas-Carrasco (2013) claim that studies in Australia, Canada, and the UK have shown a prevalence of 3–10%, and a broader set of investigations showed even a higher prevalence, 3–27.5%. Elder abuse has low reporting rates due to several factors (Abujarad et al., 2021), which makes developing new methods of screening essential.

Translating elder abuse screening tools into other languages is of utmost importance to reach other populations who might be at a higher risk. DeLiema et al. (2012) claim that low-income Latino immigrants are understudied in elder abuse research, and it is likely that their limited English proficiency, economic insecurity, cultural issues, and mistrust of authorities, among others, are factors that contribute to low reporting rates^1^. Using a door-to-door in-person interviewing system, they found that 40% of Latino elders had experienced some form of abuse within the previous year. The types included psychological aggression, physical assault, sexual coercion, financial exploitation, and caregiver neglect. Other studies claim that mistreatment is linked more closely to low income, poor social support, and poor health than to ethnicity (Hernandez-Tejada et al., 2013), which might explain why Latinos might be at a higher risk. Laumann et al. (2008) claim that Latinos in their study were less likely to report financial or verbal mistreatment when compared to other ethnic groups. These findings make it imperative to translate the tool into Spanish for the Latino community living in the United States.

Maneesriwongul and Dixon (2004) report that researchers in the 70s were already calling for a multi-step process for the translation and cultural adaptation of health-related instruments out of despair for the lack of consistency, the differences in terminology, and the lack of a rigorous methodology that existed at that time. There were several proposals in the 90s (e.g., Guillemin et al., 1993) that already reflected the multi-step process and added other recommendations, such as detailing the qualifications of the translators, the skills of the review team, and so forth. Wild et al. (2005) reiterate that a multi-step procedure is a solution to the issues brought up in the past century. Their suggested process includes: 1) preparation, 2) forward translation, 3) reconciliation, 4) back translation, 5) back translation review, 6) harmonization, 7) cognitive debriefing, 8) review of cognitive debriefing results and finalization, 9) proofreading, and 10) final report.

We can see that the translation and cross-cultural adaptation must go through several adaptations, adjustments, or refinements after each step until it reaches its final form. Although laborious, time-consuming, and costly (Beaton et al., 2000; Kalfoss, 2019; Povoroznyuk et al., 2016; Sperber, 2004), this multi-step process aims to increase the quality of the adapted instruments and, in turn, their overall validity and reliability (Beaton et al., 2007; Borsa et al., 2012; Guillemin et al., 1993; Karthikeyan et al., 2015; Mizuno et al., 2019; Tsang et al., 2017).

Hawkins et al. (2020) claim that following a rigorous translation procedure maximizes construct equivalence, minimizes threats to construct validity and generates qualitative validity evidence for score interpretation and use in a new linguistic context. However, others think that having a good translation does not necessarily mean that it is a valid and reliable tool (Beaton et al., 2000; Hendricson et al., 1989; Scott-Lennox et al., 1999; Sperber, 2004). Translating and validating are two separate processes: “translation includes the process of transforming the questionnaire, whereas the validation process primarily covers the process of quality assessment of the translated tool” (Danielsen et al., 2015, p. 2). When both processes are done correctly, one can assert that it is a good translation and that it is as valid and trustworthy as the original (Povoroznyuk et al., 2016; Tsang et al., 2017). Quality and validation play an important role in “ensuring that the results obtained in cross-cultural research are not due to errors in translation, but rather are due to real differences or similarities between cultures” (Maneesriwongul & Dixon, 2004, p. 175). According to Sperber (2004), translation has several pitfalls that threaten validity, including using inexperienced translators, not accounting for cross-cultural inadequacies, not following all the steps of the process, not discriminating subjects of different language skills and others. Hendricson et al. (1989) also recommend using “translators conversant in the local dialect and willing to use popular idioms and syntax” (p. 965) so that the subjects understand the questionnaire without problems. Just like the original, translated instruments should be practical, easy, and quick to administer, have clear and appropriate wording, as well as a high sensitivity rate (WHO, 2008).

In this article, we will discuss the translation and cross-cultural adaptation process of VOICES, a “self-administered digital health intervention that screens, educates, and motivates older adults to self-report elder mistreatment” (Abujarad et al., 2021, p. 1469). Obtaining a quality translation and cross-cultural adaptation from English to Spanish is complicated since Latinos living in the United States could come from 20 different countries, and culture varies from country to country, including their taboos, religious sensitivities, political referents, offensive expressions, social norms, educational systems, etc. The steps of the process that we took are the following:

1. Two blind translations from English to Spanish (by translators 1 and 2)
2. A synthesized version by a revision team (not including the translators)
3. Two blind backward translations from Spanish to English (translators 3 and 4)
4. A synthesized version of the backward translations (by the revision team)
5. A conceptual comparison between the synthesized backward translation and the original (by the revision team and the research team, which includes the tool creator)
6. Adjustments to the Spanish translation
7. Cognitive interviews and/or focus groups
8. Adjustments to the Spanish translation
9. Pilot testing
10. Adjustments to the Spanish translation

The purpose of this article is to report the process involving the translation and cultural adaptation (steps 1–6 above), providing specific examples of the issues found and how they were resolved. This article will not cover the rest of the steps, which will be reported in a future article that will also discuss psychometric results.

## 2. Literature Review

For the last 30 years, several studies have used a multi-step translation and cultural adaptation process (e.g., Bullinger et al., 1998; Ehrenbrusthoff et al., 2018; Guillemin et al., 1993; Hawkins et al., 2020; Kalfoss, 2019; Mizuno et al., 2019; Povoroznyuk et al., 2016). When comparing these studies, we can see some differences in methodology but a common goal of using a multiphase procedure that involves several people. Maneesriwongul and Dixon (2004) examined 47 studies, two of which did a forward-only translation, seven did a forward-only translation with testing, 13 added a back-translation, 18 added the back translation and a monolingual test, three used a bilingual test instead, and four used both a monolingual and bilingual test. Only 25 of the 47 studies reported that conducting and evaluating the translation was their main purpose, 38 of the 47 compared versions of the instruments, 20 used a single translator, and 40 reported reliability and validity from instrument testing. A decade later, Danielsen et al. (2015) examined the translation methodology of more than 50 health-related studies (although none was about elder mistreatment) and found that 79% included forward and backward translations, 73% used translators whose first language was the target language, and 75% had a review by an expert panel. Obviously, as the years’ pass, we are refining the system.

Apart from the differences of the translation procedure already mentioned, other differences include translators rating the difficulty level of each item (Bullinger et al., 1998); translators coming to a consensus, instead of an expert panel doing that task (Ehrenbrusthoff et al., 2018; Mizuno et al., 2019); including cognitive interviews before testing (Mizuno et al., 2019; WHO, 2003); using only one translator (Pérez-Rojo et al., 2010; Scott-Lennox et al., 1999; WHO, 2003); performing differential item functioning analyses (Bjorner et al., 1998); using an idiomatic translation adapted to a particular community (Hendricson et al., 1989); and comparing the data obtained with the Spanish-speaking population with that obtained from the English-speaking population (Scott-Lennox et al., 1999). Sperber (2004) added one more step: having 30 raters (other than translators and researchers) rank the original and backward translation versions “in terms of comparability of language and similarity of interpretability” (p. S126). According to her, these brought to light a few inconsistencies that, when fixed, improved validity and reliability. Another difference worth mentioning is that while some studies advocate for the use of professional translators with high skills (Behr, 2018), other studies state that “highly educated individuals may not be culturally representative of the target population” (Guillen et al., 1993, p. 1421) and their translations are “complicated and difficult to understand for people with less education or lay panels” (Hunt, 1993, as cited in Kalfoss, 2019, p. 10). What seems to be important in a translation is the conceptual equivalence more than the linguistic equivalence (Karthikeyan et al., 2015).

Pérez-Rojo et al. (2010) performed a linguistic and cultural adaptation of two instruments created by a Canadian team that screen for elder abuse, the Elder Abuse Suspicion Index and the Social Worker Evaluation Form. They used one expert in elder abuse to do the forward translation and two experts in English to do the backward translation. Then, the research team compared the back translation with the original and used focus groups to identify further inconsistencies. Giraldo-Rodríguez and Rosas-Carrasco (2013) also developed a questionnaire to detect elder abuse in Mexico City, and they translated it into English but did not comment on the translation process, its validity and reliability, nor specify the purpose of translating the questionnaire into English.

## 3. Methodology

### 3. 1. Materials

VOICES is a self-administered, tablet-based elder abuse intervention that includes education, screening, psychoeducation, and automated motivational elements. VOICES utilizes automated text-to-speech, multimedia elements, and a digital coach to guide the user through a customized pathway to inform and help the user identify any possible elder abuse, such as neglect, abandonment, financial exploitation, emotional abuse, physical abuse, and sexual abuse. The intervention has been developed and evaluated by our group. Also, it has been tested with older adults with disabilities, including hard of hearing, low vision, and blind populations utilizing additional accessibility features (Swierenga et al., 2021).

In order to be part of this study in Spanish, subjects must also sign an informed consent, and take a pre-survey and a post-survey. A sample of these instruments and their translation into Spanish can be found in the Appendices.

The instruments translated in the present study are meant to be used with Spanish-speaking older adults living in the United States while visiting the ER or a clinic. Since 61% of the Latino population in the U.S. is of Mexican origin, according to the U.S. Census (American Community Service, 2019), we recruited several Mexican or Mexican American team members for the study (see next section).

### 3.2. Procedure

The process that involves the multi-step translation and cross-cultural adaptation is described below:

#### 3.2.1. Forward translations:

This step consisted of two independent translations from the source language (SL), English, to the target language (TL), Spanish, done by two bilingual Spanish-English individuals. To serve as translators, we chose either Mexican or Mexican American individuals whose first language was Spanish, so the translation would produce a natural, authentic product. According to Guillemin et al. (1993), “translations are of higher quality when undertaken by at least two independent translators. This allows for the detention of errors and divergent interpretations of ambiguous items in the original” (p. 1421).

Translator 1 is a female sophomore in college who was born in Mexico to Mexican parents and lived there for 12 years before moving to the United States. She maintains regular contact with Mexico and Spanish-speaking family members. She has some experience interpreting and translating legal documents on behalf of immigrants and refugees. Translator 2 is a Mexican female born and raised in Mexico who spent six years in the United States getting a Ph.D. in Spanish literature. She returned to Mexico after graduation. She has taught Spanish in the United States for three years, and she likes to write poetry in Spanish. She also has some experience interpreting and translating, though not in a medical setting.

#### 3.2.2. Synthesized version:

This step was done by analyzing and comparing both translations, as well as contrasting those with the SL. In the review process to come up with a unified version, Hawkins et al. (2020) recommend “locate words, phrases, and concepts in the forward translation that are incorrect or require changes to achieve the most accurate, and linguistically- and culturally-appropriate translation possible” (p. 5). Following Karthikeyan et al. (2015) ‘s recommendation, we used a team of experts to review both Spanish translations, discuss the discrepancies, and come up with a consensus to produce a unified Spanish version. According to Mizuno et al. (2019), this collaborative work necessary to obtain a reconciled translation reflects “the best thinking of the group” (p. 66). To avoid bias, Beaton et al. (2007) recommend that the person creating the synthesized version should be someone different from the translators.

Reviewer 1 was born and raised in Spain. After graduating from college, she moved to the United States, where she spent the second half of her life. She has a Ph.D. in Foreign Language Education and has been teaching Spanish at the college level for more than 20 years. She has several years of experience doing translations and interpretations (including in medical settings). Reviewer 2 was born in California to Mexican parents, and her first language is Spanish. She works at a university hospital as a registered nurse, and she frequently communicates with Spanish-speaking patients, including the elderly. Other members of the research study, namely, the digital tool creator and a research assistant, also contributed to the review process by clarifying some issues in the original English version.

#### 3.2.3. Backward translations:

This step consisted of two independent back translations to English using the Spanish synthesized version as the starting point. This task was done by two bilingual English-Spanish individuals. To serve as back translators, we chose individuals who were born or raised in the United States and with some experience in translation. The main purpose of the backward translation is not to render an identical text to the SL text but to produce a conceptually equivalent text. According to Beaton et al. (2007), “this is a process of validity checking to make sure the translated version accurately reflects the item content of the original version” (p. 7).

Translator 3 is a native Spanish speaker born in Mexico but raised in the U.S. He has a double major in Spanish and Political Sciences and has experience translating and interpreting in legal settings, although not in medical settings. Translator 4 is a native Spanish speaker of Mexican origin born in the U.S. She is a graduate student with a major related to medicine. She has worked as a translator and interpreter in a neighborhood clinic and lab research studies.

#### 3.2.4. Synthesized backward translation:

This step consists of comparing both back translations and coming up with a synthesized back translation. This was done by the same two reviewers that worked on the synthesized Spanish translation.

#### 3.2.5. Conceptual comparison of the back translation and the original:

This is a comparative analysis between the synthesized back translation and the original instrument. This comparison typically reveals conceptual mismatches and inadequate lexicon use in the TL text. This work was done by the two reviewers, the tool creator and the research assistant.

#### 3.2.6. Adjustments to the Spanish translation:

The aforementioned comparison revealed a few adjustments that needed to be made to the TL text. This work was done by the review team.

## 4. Results

On a 7-point scale where 1 was unacceptable and 7 excellent, the reviewers rated the forward translations as 6 (Very good) and the back translations as 7 (Excellent). Reviewers met multiple times to discuss the problematic areas of all translations, and they also met with the research team to clarify issues with the original English version and to come to a consensus regarding some issues raised by the back translations. The purpose of these meetings is to verify that both *adequacy* and *equivalence* were reached in the translation and cross-cultural adaptation process. Povoroznyuk et al. (2016) define equivalence as a construct that “stipulates the degree to which the output is well-formed (….) [and] adequacy refers to the extent to which the output communicates the information present in the reference translation” (p. 144). Regarding equivalence, Beaton et al. (2000) mention four different types: *semantic equivalence* refers to the fact that the words in the source instrument mean the same as those in the translated instrument, *idiomatic equivalence* refers to the accurate translation of idioms and colloquialisms, *experiential equivalence* refers to daily tasks that might not apply in a different culture, and *conceptual equivalence* refers to the differences between concepts from one culture to another.

Upon reviewing the translations of all instruments (digital tool questions, informed consent, and pre-and post-surveys), the issues that were discussed by the review team were analyzed and classified *a posteriori* into these seven categories, which will be discussed in the next section:

1. Word format depending on the gender of the subject answering the questionnaire
2. English interference
3. Typical spelling errors by heritage learners and native speakers
4. Problems originating in the source text
5. Cultural issues
6. Grammar and vocabulary issues
7. Register

Some of the lexical clarifications in the following section refer to the definitions provided by Real Academia Española (RAE), an official institution founded in Spain in 1713 with the mission to ensure the stability of the Spanish language by applying linguistic prescription aimed at promoting unity and grammatical correctness.

In the report, we will use SL to indicate Source Language, T to indicate translation done by the translators, and TL to indicate Target Language or final Spanish version.

### 4.1. Word format depending on the gender of the subject answering the questionnaire

Spanish is a language where grammatical gender (masculine and feminine) affects different types of words, such as some nouns, adjectives, direct object pronouns, and articles. Since the instruments were asking questions directly to the participant, these words had to match the gender of that person. The reviewers had three options to deal with this issue: creating two separate questionnaires (one for male participants and another for female participants) or writing the word showing both genders (e.g., amenazado/ amenazada) or (amenazado/-a). Since the target population consists of elders who potentially could get confused when having too many unnecessary words in the questionnaire, the team decided to create two separate questionnaires, one for male participants and another for female participants. The text-to-speech feature as well was considered when making this decision to have the conversational tone in voice sound more natural.

### 4.2. English interference

The transfer of certain linguistic forms from one language to another is an inevitable phenomenon experienced by people who know two or more languages as translators. These are a few examples of the dominance of English over Spanish in the translators’ repertoire:

SL: depend on       T: en las que dependen    TL: de quien dependen

SL: signs of neglect     T: signos de negligencia  TL: señales de negligencia

SL: never attended school T: nunca atendió…    TL: nunca estudió…^2^

SL: check weather T: checar^3^ el clima     TL: revisar el clima

SL: receive email T: recibir mails^4^      TL: recibir correo electrónico

SL: chat with family     T: chatear^5^ con familia  TL: charlar con familiares

SL: I felt the fonts T: sentí que las fuentes    TL: creo que los tipos de letra

Depending on the participants’ level of English proficiency, it is possible that some of these loanwords might actually be more appropriate than the actual Spanish word the review team suggested. The testing phase will reveal that.

There were a few expressions related to hospitals or health issues that both translators translated literally. The reviewers believe that this is because neither of them is particularly familiar with medical translation. Here we must agree with Behr (2018) when she stated, “the more a translator knows of a given subject matter (…), the better he or she will be able to translate a given text” (p. 5):

SL: no intoxication?

T: ¿No está intoxicado?

TL: ¿No está borracho o bajo la influencia de drogas?^6^

SL: in doctor’s office     T: en la oficina del doctor TL: en el consultorio del doctor

SL: the Emergency Room T: Sala de Emergencias  TL: Urgencias

The last example showed to be problematic in the back translation. Both back translators used the term ‘Urgent Care’ when they translated ‘Urgencias’. Urgent Care is a different kind of service from the E.R. Due to this conceptual discrepancy between the back translation and the original SL text; the review team accepted ‘Sala de Emergencias’, which, although not 100% authentic, is perfectly understood by the Latino population.

Some punctuation rules are different in English and Spanish, and occasionally the translators slip. For example, there is no comma in Spanish before the last item of a list is introduced by a conjunction. Also, the comma and the period, when accompanying numbers, are reversed: the English comma for thousands is a period in Spanish and the English period for decimals is a comma in Spanish.

### 4.3. Typical spelling errors by heritage learners and some native speakers

A heritage learner is a person who was raised in a home where the TL is spoken and speaks or at least understands that language, even though the official or main language of the country is a different one. Heritage learners and some native speakers make certain types of spelling errors due to little or lack of reading experience and the phonetic similarities between some words. For instance, the equivalent in English would be confusing ‘there’ with ‘their’ or ‘it’s’ with ‘its’. Here are some examples that need to be corrected by the reviewers:

SL: have you relied on people    T: ¿a dependido  TL: ¿ha dependido

SL: contribute to         T: contribuir ha  TL: contribuir a

SL: because              T: por que        TL: porque

Another issue involves confusing words that look similar but actually mean different things, like ‘deber de’, which conveys probability, and ‘deber’, which conveys recommendation or obligation, or confusing ‘any way’, which means ‘any manner’ with ‘anyway’, which is an adverb that means ‘regardless’:

SL: What should you do if    T: ¿Qué debe de hacer si  TL: ¿Qué debería hacer si

SL: you do not have to       T: no debe de           TL: no tiene que

SL: mistreated in any way?    T; de cualquier manera?   TL: de alguna manera?

Diacritic accents in Spanish are problematic for heritage learners but also for many native speakers. Reviewers found these translation errors that were not flagged as incorrect words by the translators’ word processor because they are actual words in Spanish, they just have a different meaning:

incomodo (I disturb) / incómodo (uncomfortable),

abandono (neglect, abandonment) / abandonó (he/she abandoned or left)

sabana (savanna) / sábana (bed sheet)

medica (he/she/formal you medicates) / médica (medical, female doctor)

cortes (cuts, you may cut) / cortés (courteous, polite)

esta (this) / está (is)

porque (because) / por qué (why)

mas (but) / más (more)

cuanto (all the) / cuánto (how much)

participe (I/formal you/he/she might participate) / participé (I participated) / partícipe (part, collaborator, beneficiary)

que (that) / qué (what)

### 4.4. Problems originating in the SL text

The reviewers found some issues in the translations and tracked the cause of the problem to the SL text. The reviewers had to meet with the tool creator and research assistant to clarify the intended meaning of these issues.

In these examples, the lack of referents for certain pronouns caused ambiguity, apart from the difficulty of translating pronouns without knowing the gender of the noun they refer to. Once the referents were clarified, the reviewers decided to add them to the TL text to avoid any possible misunderstandings. The addition is a common technique in translation and totally justified in these cases to make the TL text more comprehensible:

SL: if any of these might

T: alguno de estos podría

TL: si alguna de estas razones podría

SL: which of the following do you

T: ¿…cuáles de los siguientes?

TL: de los siguientes aparatos, ¿cuáles…?

SL: you fear it’s getting worse

T: teme que empeore

TL: teme que la situación empeore

In the following example, we found that a missing comma between two adjectives in the SL text (which might be correct in certain circumstances in English) was also missing in both forward translations. Upon close inspection, the reviewers noticed that those two adjectives belonged to two different categories according to the English royal order of adjectives^7^, and that is why the comma was absent. However, there is no such royal order in Spanish, so a comma needs to be inserted. To clarify the long resulting sentence, the expression ‘ya sea’ (‘either, that being’) was added:

SL: in any federal, state, or local civil, criminal, administrative, legislative, or other action, suit or proceeding

TL: en cualquier acción, juicio o procedimiento federal, estatal o local, ya sea civil, penal, administrativo, legislativo o de otro tipo

The reviewers encountered a sentence with the word ‘fake’ in the SL when describing signatures. The translators translated it literally, ‘falsas’, but upon discussion with the researchers, the review team thought the term ‘forged’ was more accurate in English. Thus, the term ‘falsificadas’ was used instead.

In this example where the participants need to rate their confidence in their ability to use new technology, reviewers felt that the wording in the scale could be misinterpreted by participants, since ‘confiado’ not only means ‘confident’ but also ‘trusting’, in Spanish. The reviewers thought it could be possible that participants think they are being asked if they trust new technology. Thus, the reviewers decided to use the word ‘capable’ (‘capaz’) in the rating scale:

SL: very confident    T: muy confiado    TL: me siento muy capaz

### 4.5. Cultural issues

Gender bias was found in the Spanish translations clearly marked in the endings of professions. For example, when the word ‘nurse’ appeared in the source text, both translators chose ‘enfermera’ (‘female nurse’). Even though traditionally, this job has been performed by women and still today, there are more female nurses than male nurses; it is imperative to avoid this type of language bias nowadays. The English version already showed modern adaptation to 21^st^-century preferences with the pronoun ‘they’ and the verb in the plural in the next clause instead of ‘he/she’ and a verb in the singular, like it was common a few years ago. Our options included: 1) using both formats ‘enfermero/enfermera’ or ‘enfermero/-a’, skipping the pronoun of the following clause (since subject pronouns are not mandatory in Spanish), and writing a verb in singular; or 2) using the plural of a mixed group ‘enfermeros’, skipping the pronoun, and writing a verb in the plural. The reviewers selected the latter, even though the plural of a mixed-gender group looks exactly like the masculine plural ‘enfermeros’ (‘male nurses’). Although some concerns about this form to refer to mixed gender groups have been raised by feminist groups and the LGBTQ+ community, RAE has not yet accepted any of the proposed alternatives, such as ‘enfermeres’, ‘enfermerxs’ or ‘enfermer@s’:

SL: my mistreatment to my nurse and consider the assistance they have to offer

T: mi maltrato a mi enfermera y considerar la ayuda que me ofrezcan

TL: mi maltrato a mis enfermeros y considerar la ayuda que me ofrezcan

Something similar happened with ‘caregiver’. Interestingly, both translators used the masculine form ‘cuidador’, one translator exclusively, and the other translator alternating it with the feminine form ‘cuidadora’, even though this is also a job traditionally performed by women. The reviewers opted to use the plural again:

SL: hurt by a caregiver

T: agredida por su cuidador

TL: lastimada por sus cuidadores

Another example can be seen in the words ‘homemaker’ and ‘housekeeper’. These words in English show no specific gender. In Spanish, however, the most common translations are ‘ama de casa’ (‘female homemaker’) and ‘ama de llaves’ or ‘empleada doméstica’ (‘female housekeeper’). Although RAE admits both genders for the homemaker, it only recognizes the feminine format for a housekeeper. The reviewers decided to use the technique of omission for these two words since there was a third expression afterwards (‘chore person’) that expressed the same concept and had no gender markers in Spanish:

SL: homemaker, housekeeper or chore person

T: ama de casa, ama de llaves o persona que hace tareas domésticas

TL: persona que haga las tareas domésticas

The following is an example of what Guillemin et al. (1993) call experiential equivalence. In Spanish-speaking countries (except in Puerto Rico), students can attend Law School, Medical School, and other graduate programs right after high school. Taking into consideration “the relevance of original instrument concepts and domains in the new culture” (Borsa et al., 2012, p. 430), the reviewers decided to replace the question regarding those graduate school degrees with ‘Otro tipo de diploma’ (‘Other type of degree’). This way, Puerto Rican participants could add their Law or Medical degree here. As a bonus, this solution would also allow any participant to add any degree offered in their country that does not exist in the United States:

SL: Professional school degree (e.g., MD, DDS, DVM, JD)

TL: Otro tipo de diploma

When asking participants about a specific product or practice that only exists in the United States, there is no need to translate it into Spanish because subjects will probably recognize the English term and will not be familiar with the term the translator makes up to compensate for this gap. This type of problem was also present in Goerman et al. (2018)’s study with the term ‘home schooling’, whose translation was not understood by their participants, but the original English term was easily recognized. For this type of problem, the reviewers decided to leave the term in the SL:

SL: Medicare fee for service, Medicare managed care

T1: Cuota de Medicare por servicio, Atención administrada por Medicare

T2: Tarifa por servicio Medicare, Asistencia Medicare para manejo de salud

TL: Medicare fee for service, Medicare managed care

### 4.6. Grammar and vocabulary issues

The passive voice can be difficult to translate. In this example, both translations were too literal and not natural. Eventually, the reviewers modified it, keeping the passive voice in a clause in the subjunctive mood and replacing the verb ‘llevar’ (‘to lead’) with the verb ‘ocasionar’ (‘to cause’):

SL: may also lead to you being discharged to a long-term care facility

T1: puede llevarlo a ser dado de alta a un centro de…

T2: puede llevar a que le den de alta a un centro de…

TL: puede ocasionar que usted sea transferido a un centro de…

In this example, using a clause in the subjunctive was again the solution to a translation issue. The phrase *‘make us feel anxious’* would produce in Spanish an expression with two first-person plural pronouns, *‘hacer**nos* *sentir**nos* *ansiosos’*. Although grammatically correct, Translator 1 found it “awkward” and “was not sure who ‘us’ referred to”, so she changed the pronoun from ‘us’ to ‘you’ to avoid two identical pronouns. Translator 2 deleted one of the pronouns, which is incorrect. By using the subjunctive, the reviewers avoided the double pronoun issue but still maintained the first-person referent:

SL: make us feel anxious

T: hacerle sentirse ansioso

SL: puede hacer que nos sintamos ansiosos

This example also involves a pronoun, the Spanish pronoun “lo” (which can be translated by ‘him’ –or ‘them’ in gender-neutral language) and by ‘formal you’. The reviewers decided to change the sentence to the plural:

SL: someone who has a medical condition that makes them be aggressive

TL: alguien que tiene una condición médica que lo hace ser agresivo

Final TL: personas que tienen una condición médica que las hace ser agresivas

There is an apparent agreement mismatch in the following example that many translators try to avoid or do not know how to handle. The issue involves two nouns (one masculine singular and another feminine plural) followed by an adjective, which needs to go in the masculine plural form. Since neither of the nouns is masculine plural, it looks weird, even though it is grammatically correct. To avoid awkwardness, the reviewers decided to turn the passive voice sentence into an active voice sentence:

SL: when your money or belongings are stolen or go missing

TL: cuando le roban su dinero o sus pertenencias, o desaparecen

Prepositions are also problematic when translating from one language to another. In the following examples with dangling prepositions, translators decided to eliminate them altogether, which is incorrect:

SL: not being familiar with or having difficulty handling financial matters

T: no está familiarizado o tiene dificultades manejando asuntos financieros

TL: no está familiarizado con o tiene dificultades manejando asuntos financieros

SL: Which of the following do you own or have easy access to?

T: ¿Cuál de los siguientes posee o tiene acceso fácil?

TL: De los siguientes aparatos, ¿cuáles posee o a cuáles tiene fácil acceso?

SL: I’m concerned about this and the effects it might be having on you

T: Me preocupa esto y de lo que puede estar sufriendo usted

TL: Estoy preocupado por esto y por los efectos que esto pueda ocasionarle

Sometimes, a word in English has two different meanings in Spanish, and it is the context or other clues that clarify the intended meaning. In these two examples, the adjectives ‘aware’ and ‘attentive’ have different meanings if they go with the verb ‘ser’ or ‘estar’, both of which mean ‘to be’. For example, ‘ser consciente’ means ‘to know, to have something present’, and ‘estar consciente’ means ‘to be awake, not passed out’; ‘ser atento’ means ‘to be courteous’, but ‘estar atento’ means ‘to pay attention’:

SL: I hope you are more aware of

T: Espero que esté más consciente de

TL: Espero que sea más consciente de

SL: ‘Feeling: …nervous, determined, attentive, etc.

T: Se siente: …nervioso, determinado, atento, etc.

TL: Estoy: …nervioso, decidido, atento, etc.

In English, ‘injury’ can refer to either open or non-open wounds. The correct translation for this word would be ‘lesión’, which can encompass both meanings. However, most people use ‘lesión’ for non-open wounds and ‘herida’ for open wounds in real life. One of the translators used ‘herida’ and the other ‘lesión’. The reviewers decided to use both words in Spanish to cover all possible meanings of ‘injury’ in the source text

The part of the schooling system called ‘high school’ has different names in different Spanish-speaking countries, as Goerman et al. (2018) also found, such as Escuela Superior (Puerto Rico), Preparatoria or Escuela Secundaria (Mexico), Instituto (Spain), bachillerato, etc. In their study, they decided to leave the English expression next to the Spanish term to make sure it would not create confusion. We decided to do the same.

The combination ‘how + adjective’ is quite common in English, but in Spanish, the equivalents ‘cuán + adjective’ or ‘qué tan + adjective’ are either formal, obsolete, or not common in many Spanish-speaking countries. The reviewers finally decided to turn the question into a statement that means ‘indicate your level of satisfaction’:

SL: how satisfied were you with the following statements?

TL: indique su nivel de satisfacción con las siguientes declaraciones

When talking about people who participate in a research study, the terms ‘subject’ or ‘participant’ are used, which would translate to ‘participante’ in Spanish –as someone who actively participates in an event. However, one of the translators used the word ‘partícipe’ on two occasions. ‘Partícipe’, however, refers to a person who contributes to something, who shares in something^8^, or who has a part in the benefits, which is not the meaning intended here.

A word that was somewhat problematic was ‘sleep’ because although it refers to the act of sleeping (‘dormir’), the word ‘sueño’ is typically used in Spanish, even though this word also means ‘dream’. Both translators alternated between the terms ‘dormir’ and ‘sueño’, and so did the reviewers, who finally decided on these combinations:

SL: sudden or unusual changes in behavior, eating habits, or sleep

TL: cambios repentinos o inusuales de comportamiento, hábitos alimenticios o sueño

SL: poor sleep

TL: problemas al dormir

SL: Lose more sleep

TL: Perder más sueño

SL: You want to avoid worsening sleep problems

TL: Quiere evitar que sus problemas de sueño empeoren

### 4.7.- Register

There are two ways to address someone in Spanish, formally and informally. This register is evidenced not only in pronouns but also in adjectives and verbal conjugations. Since this digital tool will be used by older adults, the formal register is the most adequate. Despite the fact that the formal register was present in the entire translation from the beginning, the reviewers found a couple of instances where the informal register slipped through and needed to be fixed.

The reviewers also found colloquial expressions typical of the oral register but not consistent with the formal written register found typically in medical instruments. Those needed to be modified:

SL: Feel free to ask the research assistant at any point

T: Puede preguntarle al asistente cuando sea

TL: Puede preguntarle al asistente de investigación en cualquier momento

SL: when possible

T: lo más que sea posible

TL: cuando sea posible

## 5. Discussion

The multi-step process for translating and culturally adapting health-related instruments that started in the 70s has improved with the years and has become the norm. This process in the present study has proved to be a detail-oriented and comprehensive procedure that yielded an adequate and equivalent product when compared to the original. A thorough analysis of the forward and the backward translations of the VOICES digital tool to screen for elder abuse and other medical instruments associated with the research study provided insightful discoveries that helped refine the synthesized Spanish translation. The issues that arose during the team discussions of the forward translations were grouped using a taxonomy that originated from the actual data. The categories included 1) the gender of the person taking the questionnaire, 2) English interference, 3) typical spelling errors, 4) problems originating in the source text, 5) cultural issues, 6) grammar and vocabulary issues, and 7) register. Some of these categories are similar to those found in other studies (Hawkins et al., 2020; Kalfoss, 2019).

The category that caused more discrepancies was Grammar and vocabulary, which was expected. In terms of grammar, the issues we encountered involved pronouns, passive voice, prepositions, and gender/number agreement. Lexicon was sometimes problematic in cases of words that have two equivalents with different connotations (such as ‘injury’: ‘lesión/ herida’) or different terms in different countries (such as ‘Escuela Superior’, ‘Escuela Secundaria’, ‘Bachillerato’, ‘Instituto’). Sometimes the solution was to include both words in the TL text, and sometimes we chose to add the English equivalent in parenthesis next to the term. We also encountered homonyms (words having two or more meanings), which were clarified by context or surrounding words. It is worth mentioning the interesting case of the royal order of adjectives that exists in English but not in Spanish, which caused a punctuation error.

Both forward translations showed a gender bias, which was an unexpected finding. Even though binary grammar gender languages like Spanish are more susceptible to explicitly show cultural gender bias than non-binary grammar gender languages like English, it was unexpected to find it in a translation done by people born or mostly raised in the 21^st^ century. This shows how deep gender bias can be embedded in a culture and how it passes from generation to generation without speakers hardly noticing it. Our translation and cultural adaptation process remediated this problem and hopefully set an example for future translations.

When comparing the final back translation with the original instrument, the review team identified 12 items with problems, which were discussed to see if there were possible conceptual mismatches. Some of them include ‘more neglected’ vs. ‘less cared for’, ‘law’ vs. ‘law enforcement’, ‘willing’ vs. ‘prepared’, ‘unknown’ vs. ‘unsure’, and ‘welt’ vs. ‘hive’. The Spanish synthesized version was adjusted accordingly once consensus was reached. The back translation shed light on one item that was somewhat interesting: ‘Urgencias’ vs ‘sala de Emergencias’ to refer to the Emergency Room. Although ‘Urgencias’ is a more authentic term, the back translation yielded ‘Urgent Care’, which is a different kind of service. The team finally decided to sacrifice authenticity in favor of clarity and chose to use ‘sala de Emergencias’, a term that the target population would recognize as well. The team also left untouched terms that only exist in English, such as ‘Medicare fee for service’ or ‘Medicare managed care’.

## 6. Conclusion

As developments in technology and medicine increase, so too increases the elder population of this planet. The U.S. Census Bureau informs us that it is more common now (compared to 10 or 20 ago) that elders are childless. They also report that about 27% of older adults who live alone are childless (U.S. Census Bureau Newsroom, 2021). Since most mistreatment is perpetrated by someone outside the immediate family (Laumann et al., 2008), elders are increasingly more vulnerable as time passes. Since 6.5% of those childless adults ages 55 and older are Hispanic, according to the same Census report, it is of utmost importance that we reach this vulnerable population by providing high-quality translations of health-related instruments that will screen for elder mistreatment.

The multi-step translation and cultural adaptation process of the VOICES digital intervention, informed consent, pre-survey and post-survey provided a very accurate final product due to the high number of revisions, the high number of people with various skills involved in the tasks of translating and revising, and the discussions held to clarify problematic issues. See Table 1 below for a graphic showing the process. If only one translator had been involved, the final product would reflect this person’s background, experience, understanding, and decision-making skills, but it would also include this person’s limitations, errors, and biases. By having a team of four translators, two reviewers, and two research members (including the tool creator), the final product is a more accurate instrument with a possibly higher degree of validity and reliability. Once a good-quality translation and cross-cultural adaptation have been achieved, the next step is obtaining data to confirm that the translation is as valid and reliable as the SL text. The future steps in this study (cognitive interviews/focus groups and pilot testing) will serve that purpose. We will disclose those findings in a separate future article.

### 6.1. Limitations of the study

This article does not include the final steps of the process: the cognitive interviews or focus groups and the pilot testing. The final Spanish translation will have to be adjusted according to the feedback from those final steps of the process. At that time, we will confirm whether the Spanish translation is adequate and equivalent and valid and reliable. We will also comment on how it compares to similar tools that have been translated into Spanish, such as the Elder Abuse Suspicion Index (EASI) (Pérez-Rojo et al., 2010).

The pilot testing will take place in New Haven, which has a 30% Hispanic population, half of which is of Puerto Rican origin^9^. Even though most of the people involved in the translation process are of Mexican origin, all Spanish speakers can understand the final Spanish version. In addition, linguistic variations have been taken into consideration when necessary. The final steps in the process will finetune the Spanish version to accommodate further regional linguistic variations and other cultural adaptations.

## Supplementary Material

1

## Figures and Tables

**Table 1:** Graphic of the translation process

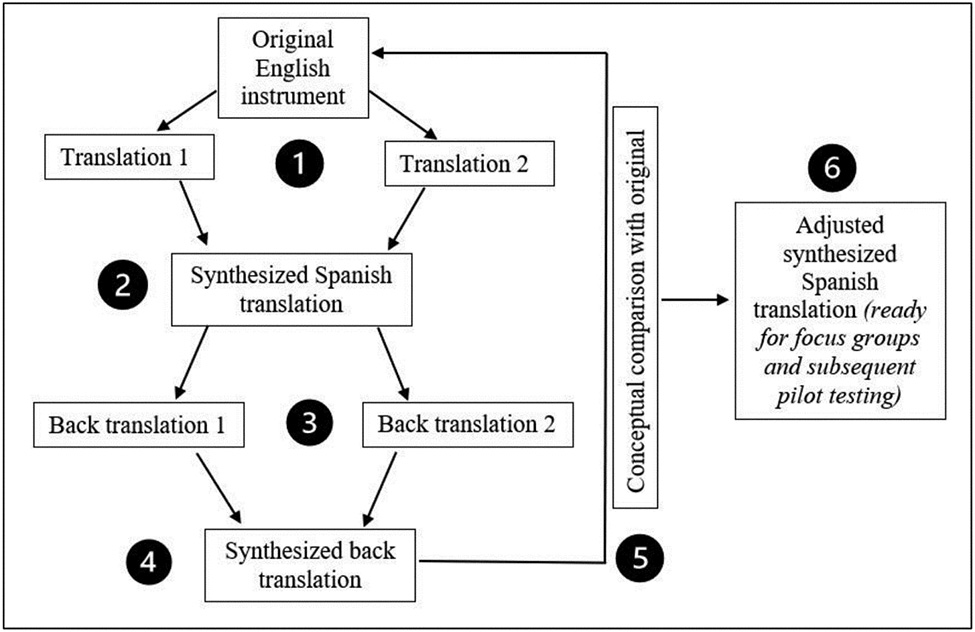

## Footnotes

1 For a more in-depth description of risk-factors in this particular community, see Parra-Cardona et al. (2007).

2 Although ‘asistió’ is an appropriate term, Goerman et al. (2018) reported misinterpretations by Spanish speakers during cognitive interviews, so they translated it as ‘estudió’ (studied). We decided to do the same to avoid possible future misinterpretations.

3 Although RAE recognizes the variant ‘checar’ off the verb ‘chequear’, it does mention that ‘chequear’ is an adaptation from the English verb ‘to check’ that has commonly used equivalents in Spanish, such as ‘revisar’ or ‘comprobar’.

4 As of today (August 4, 2021), the Real Academia Española (RAE) has not included ‘mail’ in their dictionary, even though other dictionaries have.

5 As of today (August 4, 2021), RAE has not included ‘chatear’ with the meaning of ‘chat’ in their dictionary, even though it is a popular expression and it appears in other dictionaries.

6 According to the RAE and the Free Dictionary, ‘intoxicado’ refers to a person who eats something toxic or poisonous, not to the effects of drugs or alcohol, like in the SL.

7 Retrieved on July 17, 2021, from https://www.echt-english.nl/blog/Grammar/royal-order-of-adjectives

8 According to Pons dictionary (retrieved on July 15, 2021, from https://es.pons.com/traducci%C3%B3n/espa%C3%B1ol-ingl%C3%A9s/part%C3%ADcipe)

9 According to this New Haven Independent August 12, 2021 article https://www.newhavenindependent.org/index.php/archives/entry/census_puerto_rican_flag/
