# Supplementary material for: The Process of Translating and Culturally Adapting a Digital Elder Abuse Intervention
Source: Int J Transl Interpret Stud. Author manuscript; Available in PMC 2025 Jul 24. (PMC12288910; doi:10.32996/ijtis.2022.2.1.2)
Supplement: 1 [file NIHMS2098059-supplement-1.pdf]

Appendix 1: Voices Digital Tool (Sample)
Original English version

Slide 26
Do you see a connection between your possible mistreatment and your reason for being in the Emergency Room today?
Yes No

Slide 27
Do you feel that mistreatment may have contributed to your…
- ☐ Medical issue or another health issue
- ☐ Emotional and verbal abuse
- ☐ Feeling isolated from family or friends
- ☐ Money problems, lost or stolen possessions
- ☐ Not being cared for
- ☐ None of the above

Slide 28
Do you feel that mistreatment may also contribute to…
- ☐ Wounds getting worse
- ☐ Injury
- ☐ Change in weight or not eating or drinking enough water
- ☐ Poor sleep
- ☐ None of the above

Slide 29

Do you feel that mistreatment may also contribute to…

- ☐ Feeling depressed
- ☐ Being touched in an unwanted way
- ☐ Personal reason that has not been mentioned
- ☐ None of the above

Slide 30

It sounds like being mistreated has caused you some difficulties like:

Spanish Translation

Diapositiva 26

¿Ve alguna conexión entre su posible maltrato y la razón por la que está hoy en la Sala de Emergencias?

Sí No

Diapositiva 27

¿Cree que el maltrato puede estar relacionado con…

- ☐ su problema médico u otro problema de salud?
- ☐ su abuso verbal y emocional?
- ☐ su sentimiento de aislamiento de su familia o amigos?
- ☐ sus problemas de dinero, posesiones perdidas o robadas?
- ☐ no ser atendido?
- ☐ Ninguna de las anteriores

Diapositiva 28

¿Cree que el maltrato puede también estar relacionado con…

- ☐ el empeoramiento de sus heridas?
- ☐ sus lesiones o heridas?
- ☐ su cambio de peso o no tener suficiente comida o agua?
- ☐ sus problemas al dormir?
- ☐ Ninguna de las anteriores

Diapositiva 29

¿Cree que el maltrato puede también estar relacionado con…

- ☐ sentirse deprimido?
- ☐ ser tocado de una manera no deseada?
- ☐ otra razón personal que no se ha mencionado?
- ☐ Ninguna de las anteriores

Diapositiva 30

Parece que haber sido maltratado le ha causado algunas dificultades como:

Appendix 2: Pre-survey (Sample).
Original English version

1.9 Do you live alone?
___ Yes (GO TO Q1.22) ___ No ___ Refuse

1.20 Who lives with you?

- ☐ 1… Spouse
- ☐ 2… Child
- ☐ 3… Other relative
- ☐ 4… Friend
- ☐ 5… Paid employee
- ☐ 6… Other

1.21 Total number of household members (including participant) __ ___

1.22 Which of the following best describes your current marital status:
____ Single/never married
____ Married
____ Separated
____ Divorced
____ Widow/Widower
____ Living with a partner
____ Other
____ refused

1.23 "We are interested in how well you were functioning before you came to the hospital. Which of the following best applies to you?"

Read the options in order to the patient allowing them to select one. Reread options if necessary.

- ☐ Normal activity with effort
- ☐ Cares for self, unable to carry on normal activity or to do active work
- ☐ Requires occasional assistance but is able to care for most needs
- ☐ Requires considerable assistance and frequent medical care
- ☐ Disabled, requires special care and assistance
- ☐ Patient refused

1.24 “Do you have a primary caregiver (who helps you with daily chores, personal tasks, transportation, healthcare, etc.)?”
___ Yes ___ No ___Refuse (Provide definitions of primary family caregiver designated in the SOP)

Spanish Translation

1.9 ¿Vive solo o sola?

___ Sí (Vaya a la pregunta 1.22) ___No ___Se niega a contestar

1.20 ¿Quién vive con usted?

- ☐ 1… Esposo/ Esposa
- ☐ 2… Hijo(s) / Hija(s)
- ☐ 3… Otro(s) pariente(s)
- ☐ 4… Amigo
- ☐ 5… Empleado
- ☐ 6… Otro

1.21 Número total de personas en el hogar (incluyendo al participante) __ ___

1.22 ¿Cuál de estas opciones describe mejor su estado civil actual?:

- ____ Soltero/-a
- ____ Casado/-a
- ____ Separado/-a
- ____ Divorciado/-a
- ____ Viudo/-a
- ____ Vivo con mi pareja
- ____ Otro
- ____ El/La paciente se negó a contestar

1.23 "Estamos interesados en lo bien que se desempeñaba antes de venir al hospital. ¿Cuál de las siguientes opciones describe su condición?"

Lea las opciones en orden al paciente y permita que seleccione una. Relea las opciones si es necesario.

- ☐ Hace actividades normales con esfuerzo
- ☐ Se cuida a sí mismo/-a, pero es incapaz de realizar una actividad normal o un trabajo activo
- ☐ Requiere asistencia ocasional, pero puede atender la mayoría de sus necesidades
- ☐ Requiere una asistencia considerable y atención médica frecuente
- ☐ Discapacitado/-a, requiere cuidados y asistencia especiales
- ☐ El/La paciente se negó a contestar

1.24 "¿Tiene algún cuidador o alguna cuidadora principal (alguien que le ayude con las tareas diarias y personales, transporte, atención médica, etc.)?"

___ Sí ___No ___Se negó (Proporcione definiciones de cuidador designadas en el SOP)

Appendix 3: Informed Consent (Sample)
Original English version

What are my risks if I take part in the study?
There are no medical interventions in this study, and direct medical risks are very small.

Identifying possible mistreatment can increase your risk of physical and emotional harm, or neglect. We will take all measures needed to lessen these risks. We are required to report any suspicion of mistreatment to your nurse.

The hospital, protective services for the elderly program, law enforcement, and the courts have systems in place to help keep you safe. If you feel that you are in urgent danger, your nurse and care team will create a safety plan until a more permanent solution can be found. This plan could include staying at the hospital or a safe home, or a court protective order. If you wish to disclose mistreatment, the Research Assistant will stay with you until a member of your care team comes in.

It is important to know that recognizing mistreatment may lead to harm from the caregiver or loss of the caregiver. In some cases, the caregiver may be arrested. Identification of mistreatment may also lead to you being discharged to a long-term care or skilled nursing facility.

Answering personal and sensitive questions can make us feel anxious and uncomfortable. You may be distressed while, or after using the tool. If there are any questions that you do not want to answer, or that make you upset, you can skip to the next question.

The Research Assistant will give you a brochure on resources that can offer help with mistreatment. These items can be kept with study staff until you leave the hospital if you do not want anyone else to see them.

There is a risk that the privacy of your personal information can be compromised. To lower this risk, you will be labeled by only a study number. All study-related information will be stored securely and only people who are part of the study can view it. All electronically stored data will be encrypted, and password protected.

Spanish Translation

<u>¿Cuáles son mis riesgos si participo en el estudio?</u>

No hay intervenciones médicas en este estudio y los riesgos médicos directos son muy pequeños.

Identificar un posible maltrato puede aumentar su riesgo de daño físico y emocional, o negligencia. Tomaremos todas las medidas necesarias para reducir estos riesgos. Estamos obligados a informar a sus enfermeros de cualquier sospecha de maltrato.

El hospital, el programa de servicios de protección para personas mayores, las autoridades y los tribunales cuentan con sistemas para ayudarle a mantenerse seguro/-a. Si cree que está en peligro inminente, sus enfermeros y su equipo de atención crearán un plan de seguridad hasta que se pueda encontrar una solución más permanente. Este plan podría incluir permanecer en el hospital o en un hogar seguro, o una orden de protección judicial. Si desea revelar el maltrato, el/ la asistente de investigación permanecerá con usted hasta que venga un miembro de su equipo de atención.

Es importante saber que reconocer el maltrato puede provocar daños por parte de los cuidadores o la pérdida de los cuidadores. En algunos casos, los cuidadores pueden ser arrestados. La identificación de maltrato también puede ocasionar su transferencia a una residencia de ancianos.

Responder preguntas personales y sensibles puede hacer que nos sintamos ansiosos o incómodos. Puede sentir angustia durante o después de usar la herramienta. Si hay alguna pregunta que no desea responder o que le molesta, puede pasar a la siguiente pregunta.

El/ La asistente de investigación le dará un folleto sobre recursos que pueden ofrecerle ayuda con respecto al maltrato. Estos artículos se pueden quedar con el personal del estudio hasta que salga del hospital si no desea que nadie más los vea.

Existe el riesgo de que la privacidad de su información personal sea comprometida. Para reducir este riesgo, le asignaremos un número. Toda la información relacionada con el estudio se almacenará de forma segura y solo las personas que formen parte del estudio podrán verla. Todos los datos almacenados electrónicamente estarán encriptados y protegidos con contraseña.

View publication stats
